# Network Meta-Analysis of the Safety of Drug Therapy for Cardiogenic Shock

**DOI:** 10.1155/2020/8862256

**Published:** 2020-07-31

**Authors:** Xianyong Liao, Lin Qian, Song Zhang, Xiang Chen, Jing Lei

**Affiliations:** Hospital of Chengdu University of Traditional Chinese Medicine, Chengdu, China

## Abstract

**Objectives:**

(1) To conduct a network meta-analysis of clinical drugs used for cardiogenic shock and (2) provide evidence for the selection of medication for the treatment of this condition.

**Methods:**

PubMed, EMBASE, Cochrane library, China HowNet (CNKI), Wanfang database, and Weipu database were searched using keywords Dopamine, Dobutamine, Epinephrine, Adrenaline, Norepinephrine, Noradrenaline, Milrinone, Natriuretic peptide, Recombinant human brain natriuretic peptide, Levosimendan, Cardiac shock, and Cardiogenic shock. We select literature according to prespecified inclusion and exclusion criteria and record data such as drug type, mortality, and adverse reactions.

**Results:**

Twenty-eight of 1387 articles met inclusion criteria, comprising 1806 patients who suffered from cardiogenic shock. Dopamine, dobutamine, epinephrine, norepinephrine, milrinone, recombinant human brain natriuretic peptide, and levosimendan were all commonly used in the treatment of cardiogenic shock. Milrinone was most effective at reducing mortality and had the lowest incidence of adverse reactions.

**Conclusion:**

This network meta-analysis demonstrated that milrinone was the most effective medication at reducing mortality and adverse events in patients suffering from cardiogenic shock.

## 1. Introduction

Cardiogenic shock is characterized by a decline in cardiac function leading to a significant decrease in cardiac output and insufficient effective circulating blood volume, resulting in severe acute peripheral circulatory failure. The mortality rate from cardiogenic shock ranges from 50% to 80% [1]. The most common cause of cardiogenic shock is acute myocardial infarction (AMI), accounting for 80% of cases [2]. Approximately 50% of patients with AMI develop cardiogenic shock within six hours, and 75% develop it within 24 hours [3]. The use of digitalis drugs in the treatment of cardiogenic shock is controversial. When AMI is complicated by cardiogenic shock, myocardium in the ischemic area does not bind well with digitalis, thus increasing its toxicity, suggesting it should be avoided [2, 4].

The drug of choice for the treatment of cardiogenic shock is controversial. Milrinone has been shown to affect long-term mortality from cardiogenic shock [5], and levosimendan and adrenaline have been shown to have adverse side effects, which increase potential risks and incidence of adverse reactions [6, 7]. Dobutamine has been shown to adversely increase the heart rate [8, 9] and yet is recommended by others with half of clinicians using it for treatment of cardiogenic shock [10, 11]. The purpose of this study was to conduct a network meta-analysis on the clinical effects of medications used for the treatment of cardiogenic shock.

## 2. Materials and Methods

### 2.1. Literature Review

PubMed, EMBASE, Cochrane library, China National Knowledge Infrastructure (CNKI), Wanfang database, and Weipu database were searched for articles in Chinese or English using keywords Dopamine, Dobutamine, Epinephrine, Adrenaline, Norepinephrine, Noradrenaline, Milrinone, Natriuretic peptide, Recombinant human brain natriuretic peptide, Levosimendan, Cardiac shock, and Cardiogenic shock from January 1, 2009, to December 31, 2019.

### 2.2. Inclusion and Exclusion Criteria

Articles meeting the following criteria were included: (1) randomized clinical trials related to cardiogenic shock drug therapy, (2) diagnosis of cardiogenic shock as described in the 2014 Chinese Heart Failure Guide [12], (3) cardiogenic shock as the main treatment target in the study, and (4) outcome indicators being mortality and adverse reactions. Studies were excluded if they met the following criteria: (1) nonexperimental studies such as “reviews” and “case reports,” (2) contained duplicate or low quality data or insufficient information and clinical data, (3) literature on traditional Chinese medicine and proprietary Chinese medicines for cardiogenic shock, and (4) animal experiments.

### 2.3. Data Extraction and Literature Quality Evaluation

Extracted data included the author, publication date, average age, research method, sample number, mortality rate, and incidence of adverse reactions. We evaluated bias based on evaluation criteria from the Cochrane Handbook for Systematic Reviews of Interventions including random sequence generation, whether to hide the allocation scheme, whether to use blind method, completeness of the outcome data, whether to selectively report the research results, and other sources of bias. According to the Cochrane Handbook evaluation standards, the literature is divided into 3 levels: low deviation: all meet the Cochrane Handbook evaluation standard; medium deviation: 1 undescribed Cochrane Handbook evaluation standard; high deviation: there are 2 or more items not described or 1 item does not meet the Cochrane Handbook evaluation standard.

### 2.4. Statistical Processing Methods

The network meta-analysis was conducted using ADDIS 1. 16. 8. Data were first tested for consistency using a node-split model. Where there was no statistical difference between direct and indirect comparison (*P* > 0.05), the consistency model was used. Where there was a difference, an inconsistency model was used. The stability of the analysis results of the consistency model was tested using the inconsistency model. When the inconsistency factors included 0 and the inconsistency standard deviation included 1 and the consistency model results were more stable and reliable. Various analysis models were automatically iterated based on preset parameters, and the convergence of the iterative effect was judged by potential scale reduced factor (PSRF). When the PSRF value was close to or equal to 1 (1 ≤ PSRRF ≤ 1.05), the convergence is felt to be complete, and the model is believed to have good stability, rendering the analysis conclusion more reliable. Stata 14.0 was used to create the network diagram, and funnel diagrams were made to evaluate whether the included studies had publication bias.

## 3. Results

### 3.1. Literature Review

**Figure 1:**
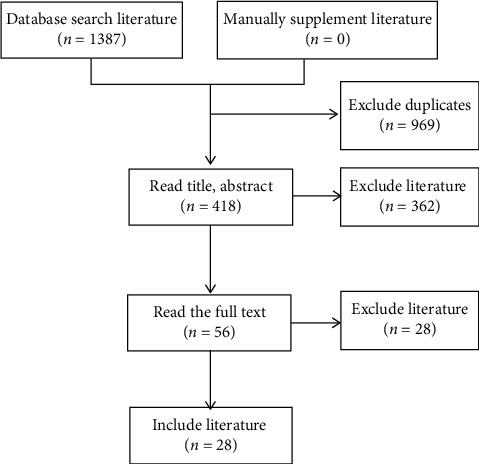
Literature screening process.

The included search terms identified 1387 articles. Using inclusion and exclusion criteria while evaluating the title, abstract, and full text of the literature, 28 articles were included, describing 1806 patients (Figure 1).

### 3.2. Basic Characteristics of the Literature

The 28 clinical studies [13–40] included were all clinical trials using medications to treat cardiogenic shock. Seven (25%) of the 28 studies were conducted before 2015, and the remaining 21 (75%) studies were concentrated after 2015. The treatment cycle and dosage of the drugs in each group were basically the same, and the difference was not statistically significant (Table 1).

### 3.3. Evaluation of Included Studies

Of the 28 studies [13–40], 7 (25%) clearly stated the method of randomization (random number table, admission order, etc.), 1 (3.5%) of the studies described the allocation concealment method, and none described the method of blinding; other sources of bias were unknown; the baseline patient characteristics of the studies were basically the same (Table 2).

### 3.4. Network Meta-Analysis Results

#### 3.4.1. Network Diagram of Included Interventions

**Figure 2:**
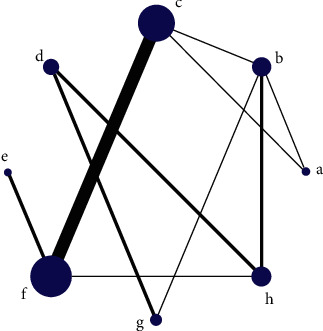
Network diagram of case fatality rate. Note: a, recombinant human brain natriuretic peptide; b, conventional treatment; c, dopamine; d, dobutamine; e, epinephrine; f, norepinephrine; g, milrinone; h, levosimendan.

**Figure 3:**
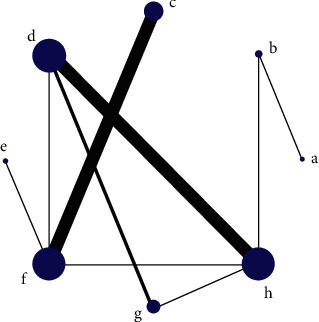
Network diagram of incidence of adverse reactions. Note: a, recombinant human brain natriuretic peptide; b, conventional treatment; c, dopamine; d, dobutamine; e, epinephrine; f, norepinephrine; g, milrinone; h, levosimendan.

Each dot in the network diagram represents a drug, and a wired segment directly connected between the two points indicates a direct comparison between the two drugs. The larger the dot, the higher the frequency of study drugs being included in the reticulation analysis. The wider the line between the two dots, the higher the frequency of comparisons between drugs (Figures 2 and 3).

#### 3.4.2. Node-Split Model Test and Convergence Judgment

Case fatality rate and incidence of adverse reactions were evaluated by node-split model method. Both *P* values were greater than 0.05, suggesting that there was no statistical inconsistency, supporting use of the consistency model for analysis. Both the consistency model analysis and the inconsistency model test of the network meta-analysis have PSRF values between 1 and 1.05, indicating that the convergence is good and the results are stable.

#### 3.4.3. Network Meta-Analysis of Case Fatality Rate under the Consistency Model

Twenty of the 1199 studies [13–32] used case fatality rate as the outcome indicator, and these were included in a network meta-analysis. According to the ranking probability map of treatment measures (Rank 8 being the best and Rank 1 being the worst), the ability of drugs to reduce fatality was as follows: milrinone > levosimendan > norepinephrine > recombinant human brain natriuretic peptide > dobutamine > epinephrine > dopamine > conventional treatment. Milrinone appeared to be the best treatment option to reduce the case fatality rate (with a probability of 44%), with levosimendan coming in second with a probability of 26%.

#### 3.4.4. Network Meta-Analysis of the Incidence of Adverse Reactions under the Consistency Model

Eighteen of 1317 studies [24–40] used incidence of adverse reactions as the outcome indicator, and these were included in a network meta-analysis. According to the ranking probability map of treatment measures, the side effect profile from best to worst was as follows: milrinone > recombinant human brain natriuretic peptide > norepinephrine > levosimendan > conventional treatment > epinephrine > dobutamine > dopamine. Milrinone appeared to be associated with the least amount of adverse reactions (with a probability of 55%), with recombinant human brain natriuretic peptide coming in second (with a probability of 32%).

#### 3.4.5. Inconsistency Model Testing

The consistency model of the two outcome indicators was analyzed and inconsistency models were used to test the stability of the results. Results demonstrated that the inconsistency factors all included 0 and the inconsistency standard deviations all included 1. This means that the results of the consistency model were stable and reliable.

### 3.5. Publication Bias

**Figure 4:**
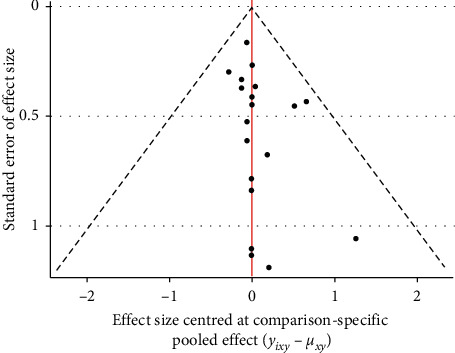
Funnel plot of case fatality rate for outcome indicator.

**Figure 5:**
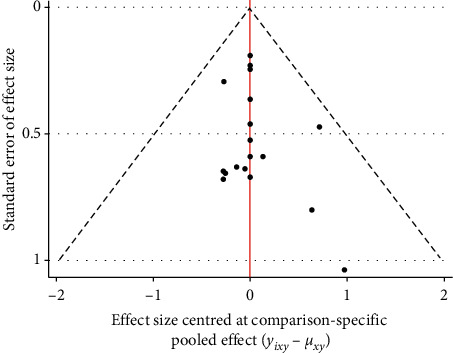
Funnel plot of incidence of adverse reactions for outcome indicators.

Funnel plots were created to identify small sample effects in the analysis. Funnel plots were created for the two outcome indicators for publication bias testing. Results demonstrated that the included studies were roughly symmetrically distributed on both sides of the funnel plot, and therefore the possibility of publication bias was felt to be small (Figures 4 and 5).

## 4. Discussion

Cardiogenic shock is a serious disease that, if not treated expeditiously and appropriately at an early stage, can have a high risk of mortality. The pathological changes of cardiogenic shock usually include two parts: one is abnormal hemodynamics and the other is insufficient perfusion of surrounding tissues. The prognosis of patients is closely related to the degree of hemodynamic abnormalities, so the rapid correction of hemodynamic abnormalities in patients with cardiogenic shock is the key to treating cardiogenic shock [41]. In clinical treatment of cardiogenic shock, blood volume is usually appropriately supplemented, and positive inotropic drugs combined with vasoactive drugs are used. For example, calcium sensitizers, levosimendan, can be combined with troponin to enhance myocardial contractility, expand coronary arteries, and improve myocardial ischemia; *β*-receptor agonist dobutamine mainly stimulates myocardial *β*1 receptors and produces a positive inotropic effect. Phosphodiesterase inhibitors milrinone can inhibit phosphodiesterase III, increase the content of cyclic adenosine monophosphate in myocardial cells [42], exert positive inotropic effects, expand blood vessels, and improve hemodynamics. Recombinant human brain natriuretic peptide with peripheral vasodilators has similar biological activity with human-derived BNP, which can dilate blood vessels, reduce heart load, and inhibit ventricular remodeling. Although many medications have been investigated for the treatment of cardiogenic shock, a direct comparison of the effectiveness of these medications has not previously been conducted. We aimed to compare previously described medications for use in cardiogenic shock with the hope of identifying preferable medications that could be administered quickly in an emergency setting.

Our study identified milrinone as being the most effective medication for reducing fatality and having the best side effect profile. Milrinone can improve the patient's hemodynamic abnormalities and hypoperfusion status, thereby rapidly improving cardiac function and correcting heart failure, Therefore, the rapid and effective application of milrinone is of great significance to save patients' lives. Levosimendan and recombinant human brain natriuretic peptide were the second most effective drugs in reducing the case fatality rate and adverse reactions. They have a positive effect on improving the clinical symptoms and prognosis of patients, and they can be used according to the patient's condition.

## 5. Limitations

Limitations of this study include lesser quality of some studies included in the analysis, small sample sizes in others, and varied lengths of treatment across studies. These factors could affect the reliability of the reticulated meta-analysis. It is hoped that larger, multicenter randomized controlled trials will provide clinical data in the future to achieve a more comprehensive understanding and evaluation.

## 6. Conclusion

In this paper, a reticular meta-analysis system is used to evaluate the difference in the efficacy of various drugs on different outcome indicators, which provides evidence-based evidence for clinical treatment, is conducive to more effective control of clinical symptoms and disease progression, and also for further clinical trials provided a reference. The analysis results showed that milrinone had the best effect in reducing the case fatality rate and the incidence of adverse reactions in patients with cardiogenic shock. Levosimendan and recombinant human brain natriuretic peptide were the second most effective drugs in reducing the case fatality rate and adverse reactions. Milrinone can improve the patient's hemodynamic abnormalities and hypoperfusion status and has the best clinical effect to reduce the patient's case fatality rate and incidence of adverse reactions. Therefore, milrinone is recommended as the clinically preferred drug for cardiogenic shock.

## Figures and Tables

**Table 1:** Included literature information form.

Include literature	Published time	Average age		Gender		Number of cases		Intervention measure	
		E	C	Male	Female	E	C	E	C
Levy et al. [13]	2011	66 ± 12	64 ± 10	21	9	15	15	0.1 *μ*g/(kg·min)^e^	0.1 *μ*g/(kg·min)^f^
Pan et al. [14]	2017	64.9 ± 12.6	64.1 ± 10.8	35	13	25	23	0.005 mg/(kg·min)^a^	3–12 mg/(kg·min)^c^
Bruno et al. [15]	2018	68 (55–79)	66 (55–77)	38	19	27	30	0.02 *μ*g/(kg·min)^e^	0.02 *μ*g/(kg·min)^f^
Zhou et al. [16]	2012	62.3 ± 1.8	60.2 ± 2.4	21	19	20	20	60 *μ*g/kg^g^	b
Pang and Zhao [17]	2011	60.2 ± 9.8	60.2 ± 9.8	28	22	25	25	75 *μ*g/kg^g^	NA^d^
Li [18]	2016	68.4 ± 11.3	70.8 ± 10.7	23	17	20	20	12 *μ*g/kg^h^	b
Chen [19]	2018	58.22 ± 5.03	60.05 ± 4.85	43	19	30	32	^f^ 0.05–0.19 *μ*g/(kg·min)	5–20 *μ*g/(kg·min)^c^
Xiong et al. [20]	2016	78.76 ± 7.17	76.77 ± 6.41	40	20	30	30	^f^ 0.05–2.0 *μ*g/(kg·min)	5–20 *μ*g/(kg·min)^c^
Tsagalou et al. [21]	2009	70 ± 9	69 ± 11	22	3	12	13	6 *μ*g/(kg·min)^h^	10 *μ*g/(kg·min)^d^
Yang [22]	2015	66.7 ± 6.5	65.3 ± 6.2	31	15	23	23	6 *μ*g/kg^h^	2.5 *μ*g/(kg·min)^d^
Zhang and Xiao [23]	2010	65.9 ± 0.2	65.9 ± 0.2	48	40	44	44	2–10 *μ*g/(kg·min)^c^	b
Lewis et al. [24]	2019	72.5 (59–81)	75 (67–83)	51	49	50	50	0.25 *μ*g/(kg·min)^g^	2.5 *μ*g/(kg·min)^d^
Pan et al. [25]	2018	68.36 ± 10.78	68.78 ± 10.72	46	34	40	40	12 *μ*g/kg^h^	b
Shen [26]	2017	46.0 ± 6.1	46.6 ± 6.3	26	22	24	24	12 *μ*g/kg^h^	^f^ 0.01–0.1 *μ*g/(kg·min)
Tan [27]	2016	56.75 ± 12.17	58.37 ± 13.24	45	35	30	50	^f^ 0.05–0.19 *μ*g/(kg·min)	10–20 *μ*g/(kg·min)^c^
Wang et al. [28]	2011	57 ± 13	57 ± 13	31	20	25	26	0.19 *μ*g/(kg·min)^f^	20 *μ*g/(kg·min)^c^
He et al. [29]	2014	63.52 ± 1.12	63.52 ± 1.12	26	21	25	22	^f^ 0.05–0.19 *μ*g/(kg·min)	10–20 *μ*g/(kg·min)^c^
Li et al. [30]	2015	58.6 ± 10.1	58.6 ± 10.1	33	27	30	30	^f^ 0.05–0.5 *μ*g/(kg·min)	1–20 *μ*g/(kg·min)^c^
Zhou and Zhou [31]	2019	67.96 + 5.92	68.13 + 5.38	93	29	64	58	^f^ 0.05–2.00 *μ*g/(kg·min)	10–20 *μ*g/(kg·min)^c^
Li [32]	2019	69.4 ± 8.7	68.1 ± 8.2	55	31	43	43	1.5 *μ*g/kg^a^	b
Lewis et al. [33]	2015	73	73	50	50	50	50	NA^g^	NA^d^
Su [34]	2019	58.5 ± 3.3	59.5 ± 3.5	38	30	34	34	^f^ 0.1–0.5 *μ*g/(kg·min)	^d^ 4.0–5.5 *μ*g/(kg·min)
Guo et al. [35]	2017	62.75 ± 2.52	62.75 ± 2.52	37	23	30	30	12 *μ*g/(kg·min)^h^	2 *μ*g/(kg·min)^d^
Peng et al. [36]	2015	59.7 ± 1.4	58.6 ± 1.2	55	53	54	54	12 *μ*g/kg^h^	2.5 *μ*g/(kg·min)^d^
Huang et al. [37]	2018	67.2 ± 3.8	68.3 ± 4.2	37	29	33	33	12 *μ*g/kg^h^	2.5 *μ*g/(kg·min)^d^
Huang et al. [38]	2018	67.65 ± 4.69	67.59 + 4.75	55	39	47	47	12 *μ*g/(kg·min)^h^	2 *μ*g/(kg·min)^d^
Chen et al. [39]	2019	68. 05 ± 6. 73	68. 46 ± 6. 47	25	15	20	20	6 *μ*g/kg^h^	25 *μ*g/kg^g^
Yang and Sun [40]	2018	61.21 ± 2.14	61.25 ± 2.44	26	24	25	25	12 *μ*g/(kg·min)^h^	2 *μ*g/(kg·min)^d^

*Note*. E is the treatment group; C is the control group. ^a^Recombinant human brain natriuretic peptide; ^b^conventional treatment. ^c^dopamine; ^d^dobutamine; ^e^epinephrine; ^f^norepinephrine; ^g^milrinone; ^h^levosimendan;

**Table 2:** Literature bias risk assessment results.

Include literature	Stochastic method	Allocation concealment	Blind method	Outcome data integrity	Selective report results	Other sources of bias
Levy et al. [13]	Randomize the code	Unclear	Unclear	Not lost to follow-up	No	Unclear
Pan et al. [14]	Unclear	Label	Unclear	Lost to follow-up, ITT analysis	No	Unclear
Bruno et al. [15]	Unclear	Unclear	Unclear	Lost to follow-up, ITT analysis	No	Unclear
Zhou et al. [16]	Admission order	Unclear	Unclear	Lost to follow-up, ITT analysis	No	Unclear
Pang and Zhao [17]	Unclear	Unclear	Unclear	Not lost to follow-up	No	Unclear
Li [18]	Unclear	Unclear	Unclear	Not lost to follow-up	No	Unclear
Chen [19]	Unclear	Unclear	Unclear	Not lost to follow-up	No	Unclear
Xiong et al. [20]	Unclear	Unclear	Unclear	Not lost to follow-up	No	Unclear
Tsagalou et al. [21]	Unclear	Unclear	Unclear	Not lost to follow-up	No	Unclear
Yang [22]	Unclear	Unclear	Unclear	Not lost to follow-up	No	Unclear
Zhang and Xiao [23]	Unclear	Unclear	Unclear	Not lost to follow-up	No	Unclear
Lewis et al. [24]	Unclear	Unclear	Unclear	Not lost to follow-up	No	Unclear
Pan et al. [25]	Unclear	Unclear	Unclear	Not lost to follow-up	No	Unclear
Shen [26]	Unclear	Unclear	Unclear	Lost to follow-up, ITT analysis	No	Unclear
Tan [27]	Unclear	Unclear	Unclear	Not lost to follow-up	No	Unclear
Wang et al. [28]	Unclear	Unclear	Unclear	Not lost to follow-up	No	Unclear
He et al. [29]	Unclear	Unclear	Unclear	Not lost to follow-up	No	Unclear
Li et al. [30]	Unclear	Unclear	Unclear	Lost to follow-up, ITT analysis	No	Unclear
Zhou and Zhou [31]	Unclear	Unclear	Unclear	Not lost to follow-up	No	Unclear
Li [32]	Random number table	Unclear	Unclear	Not lost to follow-up	No	Unclear
Lewis et al. [33]	Unclear	Unclear	Unclear	Not lost to follow-up	No	Unclear
Su [34]	Random grouping	Unclear	Unclear	Not lost to follow-up	No	Unclear
Guo et al. [35]	Random number	Unclear	Unclear	Not lost to follow-up	No	Unclear
Peng et al. [36]	Unclear	Unclear	Unclear	Not lost to follow-up	No	Unclear
Huang et al. [37]	Random number table	Unclear	Unclear	Not lost to follow-up	No	Unclear
Huang et al. [38]	Unclear	Unclear	Unclear	Not lost to follow-up	No	Unclear
Chen et al. [39]	Random number table	Unclear	Unclear	Not lost to follow-up	No	Unclear
Yang and Sun [40]	Unclear	Unclear	Unclear	Not lost to follow-up	No	Unclear

## Data Availability

The data used in the article come from clinical research, and the data used in the article can be obtained from PubMed, EMBASE, Cochrane library, China National Knowledge Infrastructure (CNKI), Wanfang database, and Weipu database. The data used to support the findings of this study are included within the supplementary information files.

## Supplementary Materials

Supplementary Materials The [zip] data used to support the findings of this study are included within the supplementary information files.
